# How Ethiopia achieved Millennium Development Goal 4 through multisectoral interventions: a Countdown to 2015 case study

**DOI:** 10.1016/S2214-109X(17)30331-5

**Published:** 2017-10-09

**Authors:** Jenny Ruducha, Carlyn Mann, Neha S Singh, Tsegaye D Gemebo, Negussie S Tessema, Angela Baschieri, Ingrid Friberg, Taddese A Zerfu, Mohammed Yassin, Giovanny A Franca, Peter Berman

**Affiliations:** a Braintree Global Health, Cambridge, MA, USA; b Department of Global Health and Population, Harvard TH Chan School of Public Health, Boston, MA, USA; c Maternal, Adolescent, Reproductive and Child Health Centre, London School of Hygiene & Tropical Medicine, London, UK; d School of Public Health, Woliata Sodo University, Woliata Sodo, SNNPR, Ethiopia; e Ethiopian Development Research Institute, Addis Ababa, Ethiopia; f Norwegian Institute of Public Health, Oslo, Norway; g Maternal and Child Health Directorate, Federal Ministry of Health, Addis Ababa, Ethiopia; h Friedman School of Nutrition Science and Policy, Tufts University, Boston, MA, USA; i Amhara Regional Health Bureau, South Wollo and Dessie City, Ethiopia; j Ministry of Health, Brasilia, Brazil

## Abstract

**Background:**

3 years before the 2015 deadline, Ethiopia achieved Millennium Development Goal 4. The under-5 mortality decreased 69%, from 205 deaths per 1000 livebirths in 1990 to 64 deaths per 1000 livebirths in 2013. To understand the underlying factors that contributed to the success in achieving MDG4, Ethiopia was selected as a Countdown to 2015 case study.

**Methods:**

We used a set of complementary methods to analyse progress in child health in Ethiopia between 1990 and 2014. We used Demographic Health Surveys to analyse trends in coverage and equity of key reproductive, maternal health, and child health indicators. Standardised tools developed by the Countdown Health Systems and Policies working group were used to understand the timing and content of health and non-health policies. We assessed longitudinal trends in health-system investment through a financial analysis of National Health Accounts, and we used the Lives Saved Tool (LiST) to assess the contribution of interventions towards reducing under-5 mortality.

**Findings:**

The annual rate of reduction in under-5 mortality increased from 3·3% in 1990–2005 to 7·8% in 2005–13. The prevalence of stunting decreased from 60% in 2000 to 40% in 2014. Overall levels of coverage of reproductive, maternal health, and child health indicators remained low, with disparities between the lowest and highest wealth quintiles despite improvement in coverage for essential health interventions. Coverage of child immunisation increased the most (21% of children in 2000 *vs* 80% of children in 2014), followed by coverage of satisfied demand for family planning by women of reproductive age (19% *vs* 63%). Provision of antenatal care increased from 10% of women in 2000 to 32% of women in 2014, but only 15% of women delivered with a skilled birth attendant by 2014. A large upturn occurred after 2005, bolstered by a rapid increase in health funding that facilitated the accelerated expansion of health infrastructure and workforce through an innovative community-based delivery system. The LiST model could explain almost 50% of the observed reduction in child mortality between 2000 and 2011; and changes in nutritional status were responsible for about 50% of the 469 000 lives saved between 2000 and 2011. These developments occurred within a multisectoral policy platform, integrating child survival and stunting goals within macro-level policies and programmes for reducing poverty and improving agricultural productivity, food security, water supply, and sanitation.

**Interpretation:**

The reduction of under-5 mortality in Ethiopia was the result of combined activities in health, nutrition, and non-health sectors. However, Ethiopia still has high neonatal and maternal morbidity and mortality from preventable causes and an unfinished agenda in reducing inequalities, improving coverage of effective interventions, and strengthening multisectoral partnerships for further progress.

**Funding:**

Bill & Melinda Gates Foundation and Government of Canada.

## Introduction

The world made steady progress towards achieving several key health Millennium Development Goals (MDGs) between 1990 and 2015. Varying country-level results based on health and non-health factors suggest that under-5 mortality decreased by two-thirds (MDG4) and maternal mortality decreased by three-quarters (MDG5).^1^ Countdown to 2015, a multiorganisational partnership now renamed Countdown to 2030, has tracked progress in women and children's health since 2003. Countdown commissioned ten country case studies (Afghanistan, Bangladesh, China, Ethiopia, Kenya, Malawi, Niger, Pakistan, Peru, and Tanzania) to assess how and why countries made progress toward achieving MDG4 and MDG5. Ethiopia was selected because data were available over relevant time periods and because strong Ethiopian in-country partners with connections to the Ethiopian Ministry of Health could undertake an independent analysis to enhance the uptake of findings.^2^ Results from the Countdown case studies informed pathways to realise the next generation of global goals and targets—the Sustainable Development Goals (SDGs).^3^

Ethiopia and six other countries for which Countdown commissioned case studies met MDG4. China and Bangladesh met MDG5, and four other countries achieved more than 75% of this target.^1^ Ethiopia reduced under-5 mortality from 205 deaths per 1000 livebirths in 1990 to 64 deaths per 1000 livebirths in 2013, with slower progress in reducing neonatal mortality (55 deaths per 1000 livebirths in 1990 *vs* 28 deaths per 1000 livebirths in 2013).^4^, ^5^, ^6^, ^7^ Global and national investments in effective community-based interventions with increased equity of coverage contributed greatly to the overall reduction in under-5 mortality in the MDG era.^1^, ^8^ Evidence also points to the interplay of political, social, and economic factors and multisectoral policies that affected child health and related MDGs.^1^, ^8^, ^9^

**Research in context**

**Evidence before this study**

The evidence base of Ethiopia's nation-wide progress in reducing child mortality and achieving Millennium Development Goal 4 has been built on studies mainly of the effects of individual-level factors such as age at marriage, fertility, birth spacing, maternal education, and socioeconomic status. At the subregional level, many studies have reported the effects of health and nutrition programme interventions as well as the effects of geographical distance from health facilities and access to health provider services. The changes in cause-specific under-5 mortality have also been examined and variations of the Lives Saved Tool (LiST) methodology applied to predict child mortality changes under different implementation scenarios of evidence-based interventions.

**Added value of this study**

We took a multidimensional approach to assess the inter-related effects of health and development policies and programmes, coverage and equity indicators, and health financing on child mortality. The LiST model provides another lens through which to assess the known contributions of measured interventions as they are related to a reduction in mortality. The synthesis and juxtaposition of these results provide a solid foundation for a more in-depth understanding of the multisectoral dynamics in improving child survival.

**Implications of all the available evidence**

The findings of this case study show the importance of broader, multisectoral approaches to improving health in low-income countries where health services are limited and where alleviation of poverty and hunger and improved water and sanitation facilities can support the achievement of child health goals. The experience of Ethiopia can inform the multisectoral pathways outlined in the Sustainable Development Goals and generate joint accountability in building effective intersectoral partnerships to achieve progress in health and development.

The following aims were set for the Countdown case study of Ethiopia: (1) assess changes in the health and non-health policy and programme environment that contributed to or detracted from progress in child survival; (2) examine the trends of health financing; (3) assess coverage trends and equity of high-impact interventions; and (4) develop estimates of selected high-impact interventions that possibly contributed to child survival using the Lives Saved Tool (LiST).

## Methods

We adapted a standard evaluation framework used to guide all Countdown case studies.^1^ The framework depicts the pathways through which the health system, health and non-health determinants, programmes and strategies, and contextual factors affect child survival.^10^ We used a set of complementary methods and a variety of nationally representative data sources, and we reviewed documents, reports, and articles. The analyses were based on structured tools emerging from the Countdown to 2015's four technical working groups: Health Systems and Policies, Health Financing, Coverage, and Equity plus the LiST modelling process.

We used three standardised tools developed by the Countdown Health Systems and Policies working group: (1) the Policy and Programme Timeline Tool; (2) the Health Policy Tracer Indicators Dashboard; and (3) the Health Systems Tracer Indicators Dashboard.^10^, ^11^ Data were derived from scientific and grey literature published between 1990 and 2014, which included peer-reviewed reports and health policy strategy documents from the Ethiopian Ministry of Health, WHO, UN agencies, and UN databases.^10^ We supplemented our findings with interviews of 68 key stakeholders from regional government health offices, two federal administrative cities, and non-governmental organisations.

Five rounds of National Health Accounts, including child health subaccounts, were compiled using a health-financing guide.^12^ We analysed longitudinal trends in health financing from total health expenditures in 1996–2011 and from child health expenditures in 2005–11. We reviewed public expenditure reviews and Ethiopian Government documents covering financing, economic development, and health, including the Health Care and Financing Strategy. All expenditure data were converted to constant US$ with the base year of 2012, using the official 1996–2011 consumer price index for Ethiopia.

We analysed trends in reproductive, maternal, newborn, and child health (RMNCH) coverage indicators (a co-coverage index) and equity measures across wealth quintiles, regions, and residential groups using Stata 14. We used the Ethiopian Demographic and Health Surveys (DHS) from 2000,^5^ 2005,^6^ and 2011^7^ to calculate coverage indicators and confidence intervals. Published results of the 2014 mini-DHS presented further trends in coverage.^13^ We used the standard DHS wealth index derived from a principal component analysis divided into five quintiles.^14^, ^15^ Classification of residences as urban or rural was based on boundaries defined by national authorities.

We used LiST (version 5.06) to estimate additional child deaths (including neonatal deaths) prevented by scale-up of health interventions, as reflected in the under-5 mortality data from 2000–11. LiST uses demographic information to model changes in child mortality from specific causes of death and changes in coverage of interventions or nutritional status.^16^ Causes of death were derived from WHO estimates,^17^ whereas mortality data were from the UN Inter-agency Working Group for Child Mortality Estimation.^4^, ^18^ All available coverage indicators were recalculated using standard Countdown definitions or LiST definitions. The default UN 2012 population data were adjusted in 2000 for child mortality estimates based on the Ethiopian Central Statistical Agency demographic projection of 1994.^19^ We used datasets from nationally representative household surveys, including the DHS, Ethiopia National Malaria Indicator Surveys (from 2007 and 2012), National Nutrition Programme Baseline Survey (2009), National Immunization Coverage Survey (2012), and Performance Monitoring and Accountability 2020. We used the reported number of beneficiaries in the Productive Safety Net Programme assessment to recalculate the balanced energy supplementation of pregnant women, and the 2008 National Baseline Assessment for Emergency Obstetric Care report to estimate service coverage at hospitals and health centres.

### Role of the funding source

The funders of the study had no role in study design, data collection, data analysis, data interpretation, or writing of the report. The corresponding author had full access to all the data in the study and had final responsibility for the decision to submit for publication.

## Results

**Figure 1:**
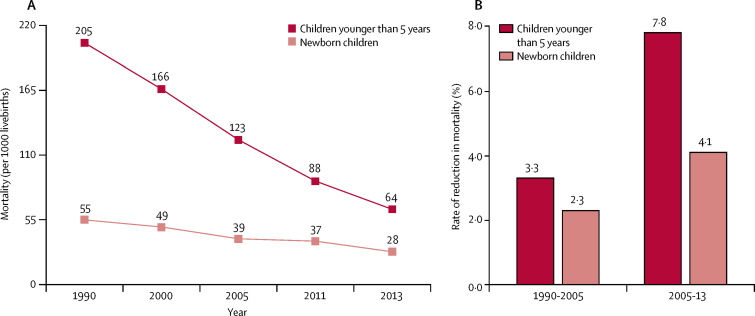
Under-5 mortality and neonatal mortality in Ethiopia, 1990–2013 Source: Ethiopia Demographic Health Survey (2000,^5^ 2005,^6^ and 2011^7^); UN Inter-agency Group for Child Mortality Estimation Report 2014.^18^

3 years before the MDG deadline, Ethiopia achieved MDG4; under-5 mortality decreased from 205 deaths per 1000 livebirths in 1990 to 64 deaths per 1000 livebirths in 2013 (a 69% reduction; figure 1). Ethiopia also recorded the fastest rate of reduction in under-5 mortality in east Africa (5·0%), exceeding that of neighbouring countries Kenya (1·5%), Sudan (2·2%), Djibouti (2·3%), and Burundi (3·1%).^18^ The annual rate of reduction for under-5 mortality increased to 7·8% between 2005 and 2013. However, newborn mortality in Ethiopia, one of the highest in the world, decreased much slower than under-5 mortality.^20^ Newborn babies still account for 45% of under-5 mortality.^4^

Trends in nutritional risk factors showed a substantial decrease in the proportion of children younger than 5 years who were stunted, underweight, or wasted between 2000 and 2014. In 2000, almost 60% of children were stunted, whereas 44% of children were stunted in 2011 and 40% of children were stunted in 2014.^13^ Similarly, the proportion of children categorised as underweight decreased from 41% in 2000 to 29% in 2011. Wasting decreased slightly between 2000 and 2011.^7^

**Figure 2:**
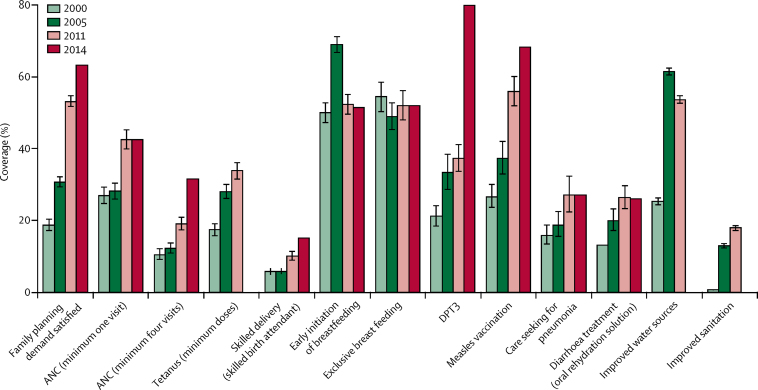
Trends in selected coverage indicators Error bars show the 95% CI. ANC=antenatal care. DPT3=diphtheria, pertussis, and tetanus vaccine.

Coverage measures are important for global monitoring because they respond more rapidly than impact measures to policy and programme interventions. Starting at very low levels of coverage in 2000, a large increase in coverage occurred in almost all measures along with improved water and sanitation by 2011 (figure 2). Assessment of coverage indicators and related 95% CI were computed for the Ethiopia DHS in 2000,^5^ 2005,^6^ and 2011.^7^ The 2014 mini-DHS raw data were not available and therefore significance levels could not be determined, but published results are included for a broad trend reference. The exceptions were early and exclusive breastfeeding, which remained constant at about 50%. Taking into account absolute values and percentage change, immunisation coverage with three doses of diphtheria, pertussis, and tetanus (DPT3) increased the most, from 21% of children in 2000 to 80% of children in 2014 (a 279% increase), with the largest change after 2011.^13^ Satisfied demand for family planning by women in reproductive age increased from 19% in 2000 to 63% in 2014. Provision of antenatal care for women who had a livebirth (a minimum of four visits) increased from 10% to 32% in the same period, but only 15% of women had a delivery with a skilled birth attendant in 2014. For these three measures, the largest upturn occurred after 2005. Key childhood services also increased between 2000 and 2011: care seeking for pneumonia increased from 16% to 27%, and diarrhoea treatment with oral rehydration solution increased from 13% to 26%. Between 2000 and 2011, the improved water indicator doubled from 25% to 54%, and sanitation improved from less than 1% to 18%, using Joint Monitoring Programme definitions.^21^ According to WHO and UNICEF Joint Monitoring Programme for Water Supply and Sanitation, improved sanitation coverage is measured as the proportion of a population with private improved pit latrines, private traditional pit latrines with slab and super structure, composting toilets, or flush or pour-flush toilets connected to sewer systems and septic tanks. The 2005 DHS added a question on shared facilities, which are currently regarded as unimproved even if they contain the above. Conversely, the proportion of the population using open defecation decreased from 82% in 2000 to 38% in 2011, whereas the proportion of the population using other forms of basic but unimproved sanitation increased from 17% to 44%. Ethiopia reported having met MDG7 target C by 2015, halving the proportion of people without sustainable access to safe drinking water and basic sanitation.^22^

**Figure 3:**
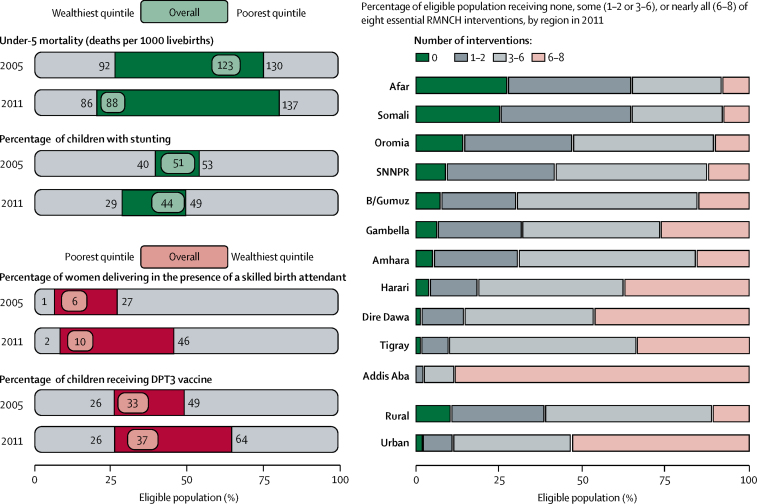
Equity of selected health outcomes and coverage indicators by wealth quintiles and co-coverage index, by residence The eight essential RMNCH interventions are Bacillus Calmette-Guérin vaccine; three doses of DPT3; measles vaccine; tetanus toxoid vaccine for mothers; vitamin A; antenatal care (minimum four visits); skilled birth attendant; and safe water. RMNCH=reproductive, maternal, newborn, and child health. DPT3=diphtheria, pertussis, and tetanus vaccine.

Data were also disaggregated by equity across wealth quintiles and area of residence between 2005 and 2011 to determine changes in child mortality, stunting, and coverage measures (figure 3). The patterns of disparities remained consistent with the poorest quintile having the lowest coverage across all indicators and highest levels of stunting and child mortality, whereas the wealthiest quintile had the highest coverage and best outcomes. Under-5 mortality increased from 130 deaths per 1000 livebirths to 137 child deaths per 1000 livebirths in the poorest quintile, whereas under-5 mortality modestly decreased in the wealthiest quintile. Most gains occurred in the middle quintiles.^23^ The wealthiest quintile had the largest decrease in stunting (from 40% to 29%), whereas stunting decreased from 53% to 49% in the poorest quintile. Only 2% of women in the poorest quintile delivered in the presence of a skilled birth attendant, compared with 46% of women in wealthiest quintile. No improvement was recorded in DPT3 vaccination coverage in the poorest quintile (constant at 26%), whereas vaccination coverage increased from 49% to 64% in the richest quintile. Disparities were highlighted by regions, where only 10% of residents in rural areas received between six and eight of the essential RMNCH interventions, whereas urban residents received nearly half of the interventions (figure 3).

**Figure 4:**
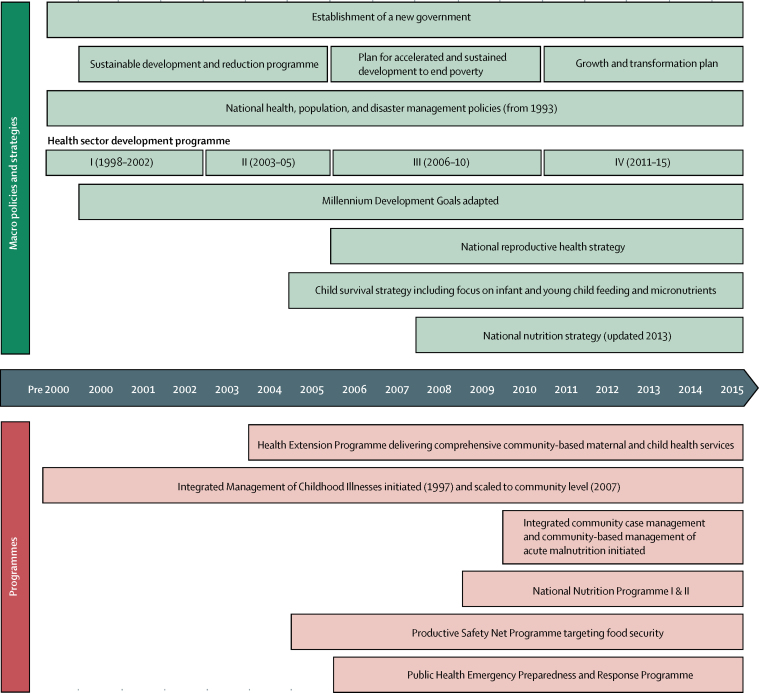
Countdown to 2015 health system and policy timeline for Ethiopia, 1990–2015 Community-based management of acute malnutrition.

Results from the Policy and Programme Timeline tool (figure 4) highlight the importance of the newly established Ethiopian Government in 1991 and multisectoral policies aimed at achieving progress in all MDGs, including child survival. The government launched three consecutive plans to reduce poverty, starting in 2000 with the Sustainable Development and Poverty Reduction Programme, followed by the Plan for Accelerated and Sustained Development to End Poverty, and the Growth and Transformation Plan (2010–15).^24^, ^25^, ^26^ Goals for agriculture, education, infrastructure, and health were incorporated into these plans to create a platform for achieving multiple MDGs. Increased agricultural productivity, coupled with the Productive Safety Net Programme, has provided assistance to more than 8 million rural residents since 2005. Assistance includes urban food subsidies to adjust for increases in food price, regulation of markets for consumer products, and labour-intensive construction projects to drive progress in poverty reduction and food security.^22^

The formulation of health policy via the Health Sector Development Plans and resultant health programmes was aligned with Ethiopia's broader development plans, while keeping resources and attention to women and children's health. The first Health Sector Development Plan was initiated in 1998 and continued as a series of 5 year programmes; the fourth Health Sector Development Plan completed its implementation in 2015.^27^, ^28^, ^29^, ^30^ With the exception of the Integrated Management of Childhood Illness, which was implemented in 1997, most policies and programmes targeting women and children's health were formulated and implemented between 2003 and 2007. A key input was the Health Extension Programme, which accelerated the expansion of health services to rural communities and changed the focus from mass campaigns and facility-based services to routine service delivery by health-extension workers. Reproductive health received some attention, but not until 2006. Clean and safe child delivery and newborn health received focus from 2008 onwards, and this focus was reinforced through the 2013 Community-based Newborn Care Initiative. Community case management of childhood illness started in 2007 and was further strengthened with integrated community case management in 2010. Nutrition received more attention in 2006, and focus on community management of acute malnutrition was expanded in 2010.

The Countdown RMNCH 11 tracer policy indicators dashboard and the health systems dashboard (appendix p 1) present a mixed picture for RMNCH. Since 2010, Ethiopia has adopted eight of the 11 policies and has met only 11% of the minimum recommended number of basic and comprehensive emergency obstetric care facilities. Ethiopia adopted and costed RMNCH plans in 2010, despite having lifesaving RMNCH commodities on its essential medicines and commodities list before 2000. Ethiopia's health workforce coverage was 2·8 skilled health-care professionals per 10 000 population in 2010, far fewer than the WHO recommended minimum of 22·8 skilled health-care professionals per 10 000 population.^31^ However, attempts were made to address this issue with a so-called flooding policy of health-care professionals in all of Ethiopia as part of the latest Health Sector Development Plans (from 2010–11 to 2014–15).^30^

Ethiopia's health sector has undergone unprecedented expansion in the past 20 years, fuelled by a substantial increase in health-sector spending. Before 2005, government health spending was biased towards hospitals and urban centres.^32^ With the implementation of the Health Extension Programme, the Ethiopian Government shifted its focus by rapidly increasing the number of health posts from 4211 in 2005 to 16 048 in 2013 and by increasing front-line health workers in all parts of the country, from 2737 in 2005 to 34 850 in 2013 (appendix p 2).^33^, ^34^ This expansion was made possible with the implementation of the Health Care and Financing Strategy (appendix p 3), focusing on increasing health funds at the local level through revenue retention, improving efficiencies, and harmonising partners towards government plans.

**Figure 5:**
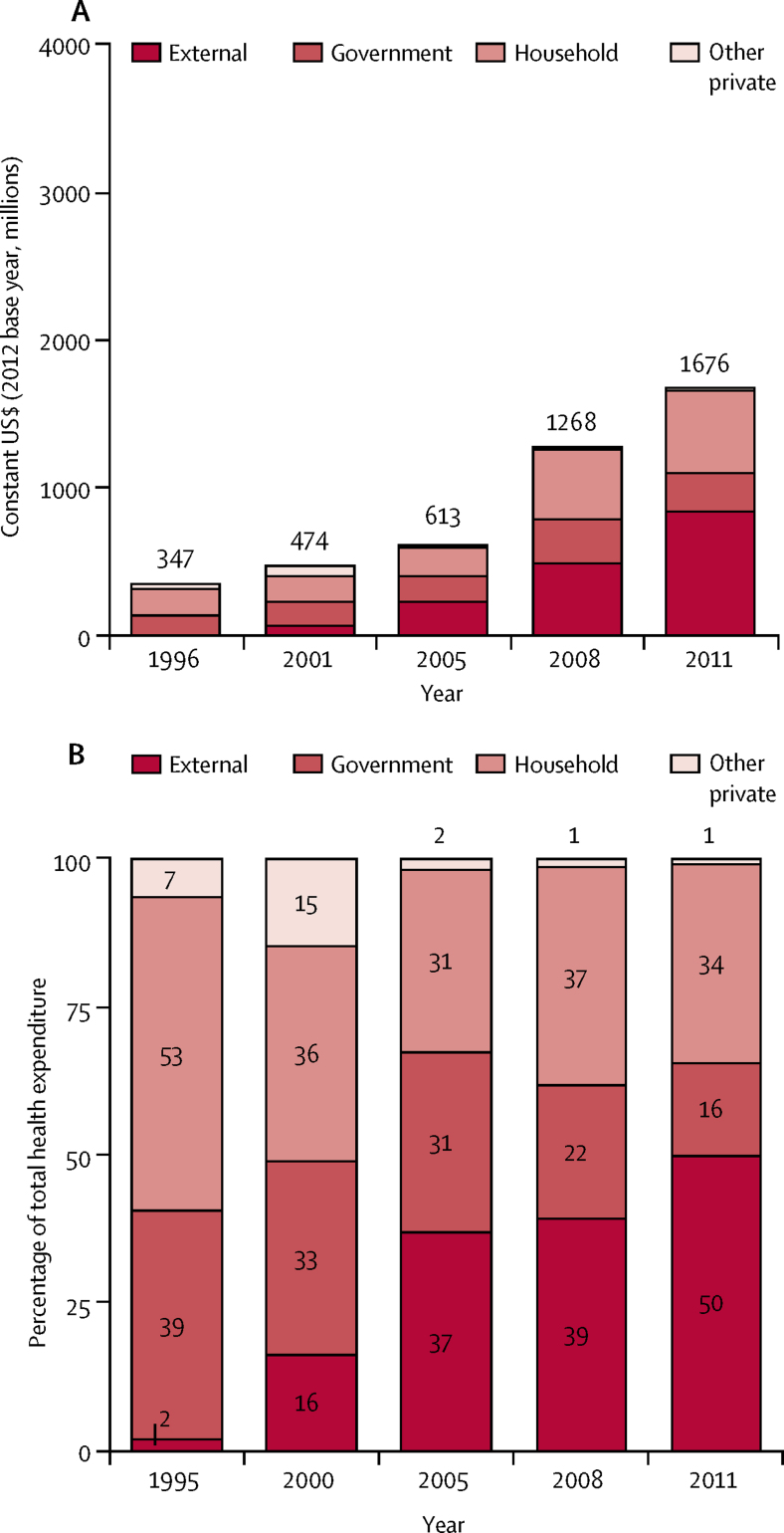
Health expenditures by funding source, 1996–2011

Total health expenditures increased seven-fold between 1996 and 2011, with a pivotal moment in 2005 when external support began to increase rapidly (figure 5). Government health spending from own resources also increased from $135 million in 1996 to $261 million in 2011; however, the percentage share of total spending by the government decreased by 24 percentage points during the same period (figure 5). Health spending as a share of total government expenditure decreased from 6·7% in 1996 to 4·4% in 2011. A greater increase in health spending occurred with a 215% increase in 2005–11, compared with a 127% increase in 1996–2005.

**Figure 6:**
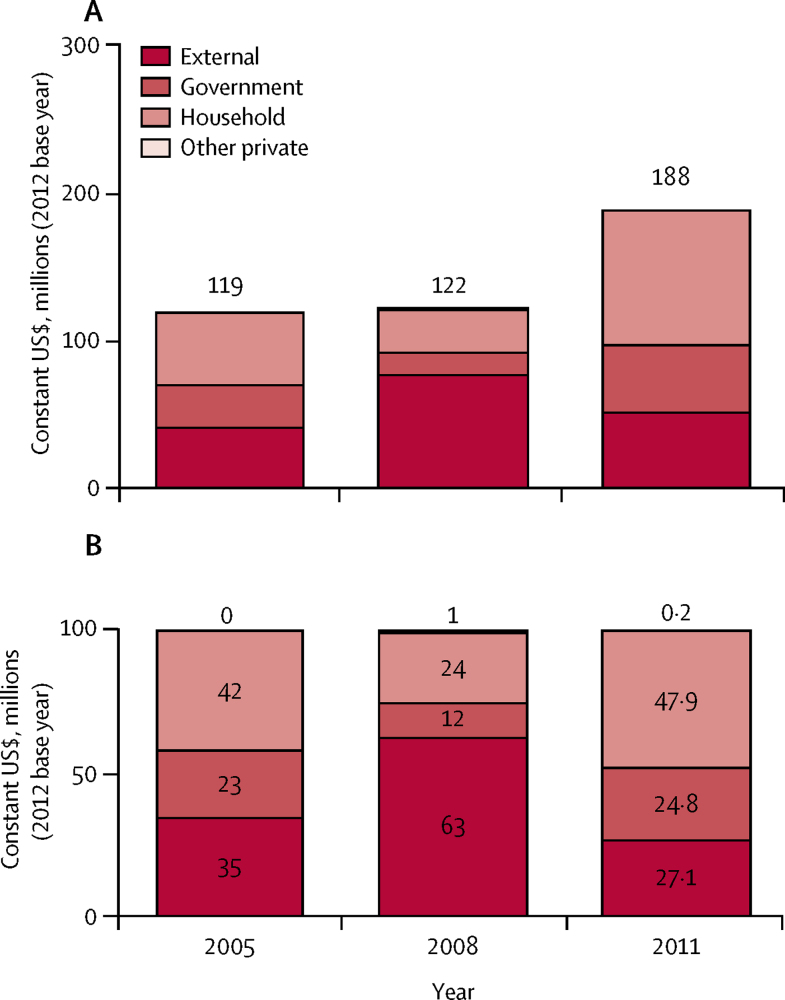
Child health expenditures by funding source

Health spending on child health programmes increased by 58% between 2005 and 2011 (figure 6); doubling the amount spent per child from $8 in 2005 to $16 in 2011. Despite this increase, households are still the main financiers, contributing 48% of child health spending by 2011.

**Figure 7:**
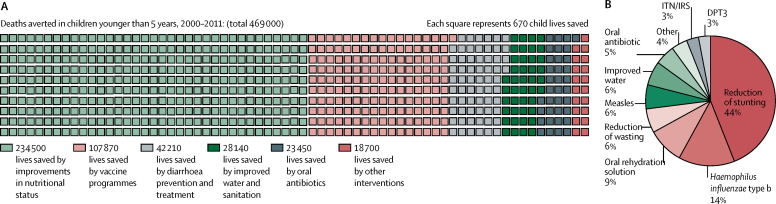
Lives saved between 2000 and 2011, as derived by the Lives Saved Tool (A) Under-5 deaths averted through health interventions and nutritional status changes, 2000–11. (B) Percentage of lives of children younger than 5 years saved between 2000–11 by high-impact interventions and changes in nutritional status. DPT3=three doses of diphtheria, pertussis, and tetanus vaccine. ITN/IRS=insecticide treated nets or indoor residual spraying.

The LiST model could explain almost 50% of the observed reduction in child mortality between 2000 and 2011. The lives of 469 000 children younger than 5 years were saved during this period as a result of Ethiopia's scale-up of high-impact interventions (figure 7). Improvements in nutritional status accounted for about half of deaths averted in children younger than 5 years, with reduced stunting accounting for 44% and reductions in severe wasting accounting for 6% of deaths averted. Scale-up of key child health interventions also provided substantial returns. Vaccination programmes accounted for 23% of deaths averted, treatment of childhood diarrhoea for 9%, pneumonia for 5%, improved water and sanitation for 6%, and insecticide-treated bednets for malaria prevention for 3% (figure 7). A rapid decrease in under-5 mortality is evident in 2005–11 (a nearly four times larger decrease than in 2000–05), with more power to explain reasons for death averted during 2005–11 (60%) than in the first half of the decade (25%).

## Discussion

Ethiopia's noteworthy improvements in child survival build on health, nutrition, and development policies that enabled synergies between a range of resources and services and supported investments in children's health. Ethiopia's progress highlights what these linkages can accomplish, despite continued low levels of health coverage, mixed results for the Countdown RMNCH 11 tracer policy indicators, and low health-care spending. After 2005, the pace of improvement accelerated and can be linked to the alignment of Health Sector Development Plans, the Child Survival Strategy, and the Health Care and Financing Strategy. The rapid expansion of health services was enabled by an infusion of external funding and the adoption of One Plan, One Budget^35^ and One Report within broader comprehensive development plans for the country.

According to the LiST model, the reduction in stunting explained about 44% of lives saved for children younger than 5 years between 2000 and 2011. Diarrhoea management and childhood vaccinations also accounted for notable effects. Ethiopia recognised at an early stage the importance of nutrition-sensitive and nutrition-specific activities to reduce stunting. The community-based nutrition programme, which incorporated the Essential Nutrition Action framework, was implemented through voluntary community health workers supervised by health-extension workers, and coverage was expanded to 228 of about 800 woredas (districts) by 2012.^36^, ^37^ The very large proportional effect of changes in nutritional status on mortality fits into the global picture as over a third of all deaths of children younger than 5 years are directly or indirectly caused by undernutrition.^38^, ^39^

Multisectoral and nutrition-sensitive approaches were based on a 2008 UN High Level Task Force on Food and Nutrition Security and the *Lancet* Series on maternal and child nutrition.^40^, ^41^, ^42^, ^43^, ^44^ As an early adopter of the Scaling Up Nutrition principles in 2010, Ethiopia had already begun addressing efficiencies in food production and food security through the National Policy and Strategy on Disaster Risk Management.^45^ In 2005, the Productive Safety Net Programme was implemented in all drought-prone woredas to provide cash or food in exchange for community service for all households identified as chronically food insecure. 6–8% of the population received support in the past decade.^46^ The coordination of this programme was led by the federal ministry of health through an interministerial National Nutrition Coordination unit.^47^

Improving water and sanitation is another health-sensitive and nutrition-sensitive intervention built on research linking sanitation (namely open defecation) to stunting, early-life cognitive deficits, and health outcomes such as height-for-age *Z* scores and infant and child survival.^48^, ^49^, ^50^, ^51^, ^52^, ^53^ In Ethiopia, open defecation has been reduced by more than half, with a large increase in traditional latrine use, but an inconsistent picture has emerged for improved sanitation.^22^ However, access to safe drinking water improved from 13% of the population in 1990 to 54% of the population in 2015.^22^

Strong synergies in health and development policies are reflected in Ethiopia's achievement of six of eight MDGs, including MDG4.^22^, ^32^, ^54^ Study findings have revealed the correlation between economic development, individual socioeconomic factors, and child health and survival.^55^, ^56^, ^57^, ^58^ Ethiopia made important strides in social wellbeing by halving the number of people living in poverty and the proportion of people living in hunger between 1990 and 2015 (MDG1). Ethiopia reported an average annual increase of 11% in real gross domestic product (GDP) from 2003–04 to 2012–13, despite high population growth.^59^, ^60^ The GDP per capita tripled from $171 in 2005, to $550 in 2013.^61^ Ethiopia's large-scale development, with growth-oriented and pro-poor sectors of education, agriculture, food security, water and sanitation, and roads, amounted to $12·7 billion in 2010–13 and accounted for 70% of general government spending in 2012–13.^61^

Our analysis has a number of limitations. First, mortality trends rely on estimates from DHS surveys, and projecting trends from birth and death registration systems are subject to inaccuracies.^62^, ^63^ Second, nationally representative coverage measures are reliant on infrequent DHS surveys, which ask a limited number of questions. Ethiopia publishes annual Health and Health Indicator Survey Reports, but data quality remains a concern.^64^ Third, health financing data from the National Health Accounts was only available from 1996–2011, so the financial landscape for the whole timeframe of the case study could not be established. Fourth, LiST modelling can only be used to estimate the reduction in mortality due to health-related interventions included in the model. Finally, we could not infer causality from the mixed-methods approach; however, our broad use of complementary Countdown methods provided a strong platform for understanding Ethiopia's achievement of MDG4.

## Conclusion

Multisectoral policies and programmes incorporating health priorities within Ethiopia's broad-based development agenda produced a powerful platform for improving child health and achieving MDG4. Ethiopia is well poised to accelerate improvements in neonatal and child health through further investments in the health system by enhancing the size and skills of the health workforce, ensuring a regular supply of drugs and equipment, and maintaining stable access to electricity and clean water in facilities. Improving the reach and capacity of the health-extension workers will enable better access to key neonatal and child interventions. Poor roads, inadequate transportation options, nomadic livelihoods, and low-quality services constrain the use of essential services and exacerbate regional and socioeconomic inequalities. Large improvements in neonatal mortality (now 45% of under-5 mortality) will depend on the provision of effective primary health-care outreach and health-care services provided in homes, communities, and health-care facilities. As access to a consistent supply of good quality interventions improves, simultaneous activation of community demand, such as the Health Development Army, can increase use and improve health gains. Creating more momentum for the education of women and economic opportunities for poor families will reduce health risks from food insecurity, poor water and sanitation, and inability to afford associated costs for health-care needs. Government investment to foster social and economic development through innovations and investments from multiple sectors could be especially beneficial in lagging regions.

The findings in the case study of Ethiopia show that child mortality can be reduced in low-income countries by leveraging connections between multiple sectors. This is especially relevant in view of the broad focus of the SDG 2030 agenda. As the SDG strategy of multisectoral engagement is implemented, new accountability and operational structures for building efficient and effective multisectoral collaboration must be identified and measured. Governments, health ministries, global health organisations, UN bodies, international non-governmental organisations, and funders will need to prioritise collective goals that go beyond their specific and programmatic interests to move towards a common, scalable vision for global health progress.

For **Countdown case studies** see http://countdown2030.org/countdown-at-the-country-level/in-depth-country-case-studies
