# Supplementary material for: How Ethiopia achieved Millennium Development Goal 4 through multisectoral interventions: a Countdown to 2015 case study
Source: Lancet Glob Health. 2017 Oct 9;5(11):e1142–51. doi: 10.1016/S2214-109X(17)30331-5 (PMC5640803; doi:10.1016/S2214-109X(17)30331-5)

THE LANCET
Global Health

Supplementary appendix

This appendix formed part of the original submission and has been peer reviewed. We post it as supplied by the authors.

Supplement to: Ruducha J, Mann C, Singh NS, et al. How Ethiopia achieved Millennium Development Goal 4 through multisectoral interventions: a Countdown to 2015 case study. *Lancet Glob Health* 2017; **5:** e1142–51.

Web Annex 1: Countdown tracer policy indicators for reproductive, maternal, newborn, and child health policy in Ethiopia

	Indicator	PRE – 2000	2001 – 2005	2006 – 2010	POST – 2010
REPRODUCTIVE HEALTH	Laws or regulations that allow adolescents to access contraceptives without parental or spousal consent	Policy adopted	Policy adopted	Policy adopted	Policy adopted
	Legal status of abortion (x of 5 circumstances)	3 (Policy partially adopted)	3 (Policy partially adopted)	3 (Policy partially adopted)	3 (Policy partially adopted)
MATERNAL HEALTH	Midwives authorised for specific tasks (x of 7 tasks)	7 (Policy adopted)	7 (Policy adopted)	7 (Policy adopted)	7 (Policy adopted)
	Maternal death notification	Policy not adopted	Policy not adopted	Policy not adopted	Policy adopted
	Maternity protection (Convention 183)	Policy not adopted	Policy not adopted	Policy not adopted	Policy not adopted
MATERNAL & NEWBORN	Postnatal home visits in the first week after birth	Policy adopted	Policy adopted	Policy adopted	Policy adopted
NEWBORN HEALTH	International Code of Marketing of Breast Milk Substitutes	Policy partially adopted	Policy partially adopted	Policy partially adopted	Policy partially adopted
	Kangaroo Mother Care in facilities for low birthweight/preterm newborns	Policy not adopted	Policy adopted	Policy adopted	Policy adopted
	Antenatal corticosteroids as part of management of preterm labour	Policy not adopted	Policy not adopted	Policy not adopted	Policy adopted
CHILD HEALTH	Low osmolality ORS and zinc for management of diarrhoea	Policy not adopted	Policy not adopted	Policy adopted	Policy adopted
	Community treatment of pneumonia with antibiotics	Policy not adopted	Policy not adopted	Policy partially adopted	Policy adopted

- Policy not adopted
- Policy partially adopted
- Policy adopted
- Not applicable

Web Annex 2: Countdown tracer indicators for health systems for reproductive, maternal, newborn, and child health in Ethiopia

	Indicator	PRE – 2000	2001 – 2005	2006 – 2010	POST – 2010
COSTED NATIONAL PLANS [adopted / costed] ✓ $	Reproductive Health	✓ $ (Data not available)	✓ $ (Data not available)	✓ $ (Policy adopted)	✓ $ (Policy adopted)
	Maternal Health	✓ $ (Data not available)	✓ $ (Data not available)	✓ $ (Data not available)	✓ $ (Policy adopted)
	Newborn Health	✓ $ (Data not available)	✓ $ (Data not available)	✓ $ (Data not available)	✓ $ (Policy adopted)
	Child Health	✓ $ (Data not available)	✓ $ (Data not available)	✓ $ (Data not available)	✓ $ (Policy adopted)
LIFE SAVING COMMODITIES IN ESSENTIAL MEDICINE LIST	Reproductive Health Commodities [x of 3]	3/3	3/3	3/3	3/3
	Maternal Health Commodities [x of 3]	3/3	3/3	3/3	3/3
	Newborn Health Commodities [x of 4]	2/4 (Policy partially adopted)	2/4 (Policy partially adopted)	2/4 (Policy partially adopted)	4/4 (Policy adopted)
	Child Health Commodities [x of 3]	3/3	3/3	3/3	3/3
SKILLED HEALTH PROFESSIONALS	Density of Skilled Health Professionals [per 10,000 population]	Data not available	6.0	2.8	Data not available
NATIONAL AVAILABILITY OF EmOC* SERVICES<br>* Emergency Obstetric Care	[% of recommended minimum]	Data not available	Data not available	11%	Data not available

- Policy not adopted; no. of health professionals does not meet any benchmarks (below 22.8 per 10,000 population); national availability of EmOC facilities is below 50% of the recommended minimum
- Policy partially adopted; no. of health professionals meets some benchmarks (between 22.8 - 41.0 per 10,000 population); national availability of EmOC facilities is between 50% and 99.9% of the recommended minimum
- Policy adopted; no. of health professionals meets all benchmarks (at least 41.1 per 10,000 population); national availability of EmOC facilities meets the recommended minimum
- Data not available

Web Annex 3: Health infrastructure and health workforce scale-up

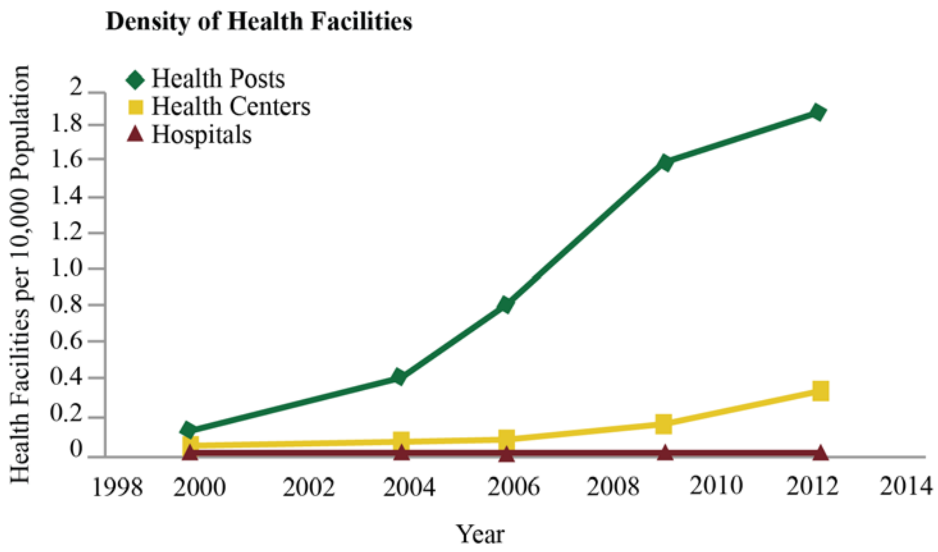

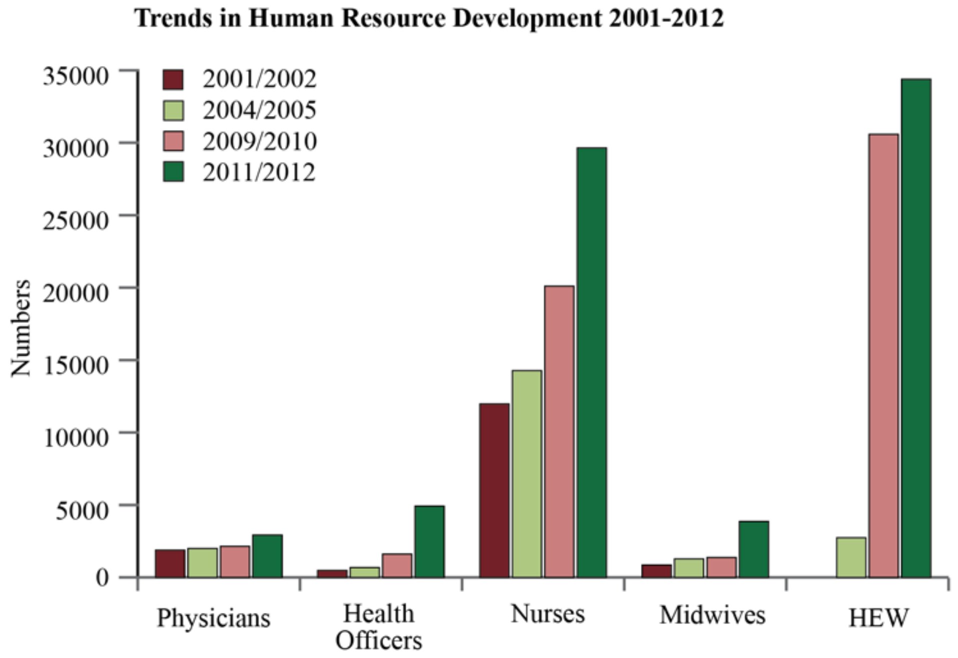

Web Annex 4: 1998 Health Care and Financing Strategy

The Health Care and Financing Strategy (HCFS) provided a clear direction to implement health financing mechanisms classified as the *first generation reforms* that focus on ways to improve resource generation and a more efficient and effective use of health resources; and *second generation reforms*, a new initiative in Ethiopia to minimize financial barriers to health services by risk-pooling and reduce payment of health services at the point of use. The latter approach introduced community-based health insurance for the informal sector and social health insurance for the formal sector. The latter is still yet to be implemented. Below are the health financing mechanisms implemented under the HCFS. Figure WA1 illustrates the health care financing timeline for Ethiopia.

User fee (revenue) retention: Ethiopia has a long history charging user fees for some public health services. Prior to the strategy, such fees were allocated up to the central treasury. This left little incentive for health facilities to collect such fees if the same amount of funds were allocated no matter the percent collected. For public health facilities to retain their own revenue has empowered them to address service delivery gaps in terms of supplies and facility maintenance and renovation. This has helped health facilities to ensure availability of pharmaceutical supplies and infrastructure like water lines, electricity, and expansion or renovation of service rooms.

Fee waiver system: Based on third-party payment principles, where health facilities will provide services free for pre-identified beneficiaries and be reimbursed by the government. This waiver scheme was introduced to allow the poorest of the poor to access medical services while alleviating the burden of paying the already subsidized user fees for certain health services.

Exempted health services: Health services that are rendered free of charge to all citizens irrespective of their level of income. Exemptions are mainly given to encourage the utilization of particular preventive, curative, or public health services. Exempted services identified are treatment of tuberculosis, leprosy, malaria, HIV/AIDS, and the provision of delivery, family planning, antenatal care, postnatal care, and expanded programme for immunizations (EPI) services.

Establishment of health facility governing body: Greater responsibility, authority, and accountability in managing service delivery and retained revenue are given to public hospitals and health centres through their own facility management boards.

Private wing and outsourcing of non-clinical services in public hospitals: These two mechanisms are implemented to minimize the high attrition rate of health workers and provide the population with additional service options under public health facilities.

Health Insurance: Both social health insurance (SHI) and community-based health insurance (CBHI) schemes are seen as important vehicles for achieving universal health coverage in Ethiopia. The CBHI was piloted in 2011 in 13 woredas within 4 large regions (Oromia, Amhara, Southern Nations, Nationalities, and Peoples' Region, and Tigray) and is being scaled-up to more than 200 woredas. The SHI strategy was endorsed, and the national and regional authorized agencies are established and working toward full-scale implementation in the near future.

Health care financing timeline for Ethiopia 1990-2016

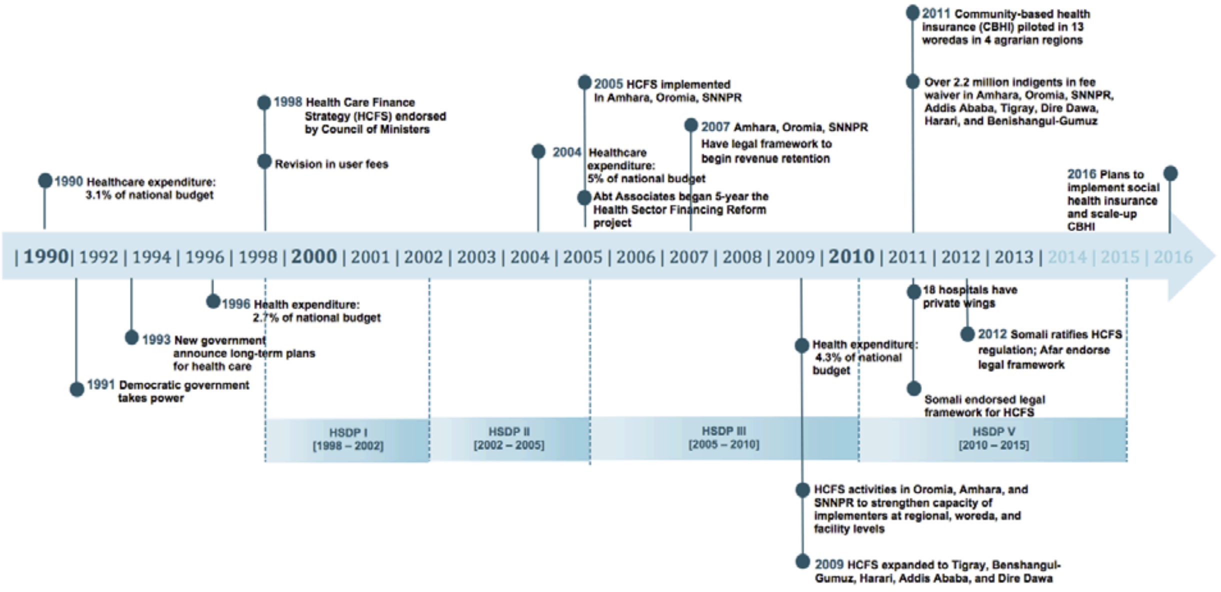

Supplement: Supplementary appendix [file mmc1.pdf]
